# PKMζ drives spatial memory reconsolidation but not maintenance

**DOI:** 10.3389/fnsyn.2025.1638371

**Published:** 2025-08-13

**Authors:** João Rodrigo de Oliveira, Janine I. Rossato, Johseph P. G. Souza, Rodrigo Orvate, Livia Carneiro, Ana Luizi Baracho, Martín Cammarota

**Affiliations:** Memory Research Laboratory, Brain Institute, UFRN, Natal, Brazil

**Keywords:** hippocampus, PKMζ, reconsolidation, spatial memory, memory maintenance, amnesia

## Abstract

Non-reinforced reactivation destabilizes spatial memory in the Morris water maze (MWM), triggering reconsolidation, a protein synthesis-dependent process that restabilizes reactivated memories. PKMζ is a constitutively active, atypical PKC isoform implicated in memory storage. However, the potential involvement of this kinase in spatial memory reconsolidation remains unexplored. We found that intra-dorsal CA1 infusion of the PKMζ inhibitor myristoylated ζ-inhibitory peptide (ZIP), but not its inactive scrambled analog scZIP, following non-reinforced spatial memory reactivation in the MWM, induced time-dependent, long-lasting amnesia in adult male Wistar rats. This effect was replicated by silencing PKMζ mRNA translation with phosphorothioated antisense oligonucleotides, but not by inhibiting the related PKCι/*λ* with ICAP, and was prevented by disrupting hippocampal GluN2B-NMDAR signaling with RO25-6981, proteasome activity with clasto-lactacystin β-lactone, and AMPAR endocytosis with dynasore hydrate. ZIP had no effect on retention when given without reactivation or after reinforced reactivation. These findings suggest hippocampal PKMζ is necessary for spatial memory reconsolidation in the MWM, but not for its passive maintenance.

## Introduction

Most animals must navigate their environment to locate food, water, and shelter, find mates, and avoid predators. Successful navigation often hinges on the effective use of external landmarks and spatial relationships to remember key locations and the routes connecting them. The hippocampus plays a critical role in encoding the spatial memories that support this allocentric navigation strategy (Rinaldi et al., 2020), a process commonly studied in rodents using the Morris water maze (MWM; Morris, 1981; Vorhees and Williams, 2006; Othman et al., 2022). In this task, animals are required to swim from various starting points along the edge of a circular pool, relying on distal cues to locate a hidden escape platform submerged just below the water’s surface. Spatial learning in the MWM is typically reflected by a decrease in escape latency over the course of training, the emergence of a lasting preference for the maze’s quadrant where the platform was located (Morris, 1984), and the development of a systematic search strategy centered on that location (Moser et al., 1993), as evidenced by a reduction in search entropy (Maei et al., 2009). However, spatial memories can become unstable when reactivated without the escape platform (Rossato et al., 2006) and, to persist, they must undergo reconsolidation (Przybyslawski and Sara, 1997; Nader et al., 2000), a restabilization process that, in the case of the MWM, is dependent on hippocampal protein synthesis and gene expression (Rossato et al., 2006; da Silva et al., 2008).

Protein kinase M ζ (PKMζ) is a constitutively active atypical PKC isoform transcribed from an internal promoter within the PKCζ gene. Its mRNA is highly expressed in neurons, particularly in dendrites, where it remains translationally repressed under basal conditions (Hernandez et al., 2003; Bal et al., 2016). Strong afferent stimulation triggers PKMζ translation, leading to a sustained increase in PKMζ protein levels and enzymatic activity at postsynaptic sites (Kelly et al., 2007). This increase can persist for hours after the induction of long-term potentiation (LTP) in hippocampal slices (Hsieh et al., 2021; Tsokas et al., 2016), and even longer *in vivo*, as observed in rodents trained on the active place avoidance or appetitive radial maze tasks (Hsieh et al., 2017). These observations, along with evidence that disrupting PKMζ signaling long after training induces amnesia across multiple learning paradigms, support the view that PKMζ is crucial for memory storage, and have led some to propose that this kinase is not only necessary but also sufficient for memory maintenance (Sacktor, 2008, 2012; Jalil et al., 2015). More recently, this hypothesis has been extended to include a putative role for PKMζ in memory reconsolidation (Crespo et al., 2012; Levitan et al., 2016; Sacktor, 2023). In this regard, it has been reported that contextual fear memory recall dynamically regulates PKMζ-mediated reconsolidation mechanisms in the basolateral amygdala (Bernabo et al., 2021), and that reconsolidation of this memory type requires PKMζ activity in the prelimbic cortex (Rodrigues da Silva et al., 2020). Furthermore, it has been shown that hippocampal PKMζ inhibition disrupts reconsolidation and leads to the erasure of reactivated object recognition memory (ORM; Rossato et al., 2019; Gonzalez et al., 2021). Interestingly, PKMζ inhibition does not impair the formation or persistence of inactive ORM (Hardt et al., 2010; Rossato et al., 2019), suggesting that, for at least some hippocampus-dependent memories, PKMζ is selectively involved in reconsolidation rather than in consolidation or maintenance. In line with this, and despite earlier claims to the contrary (Serrano et al., 2008), hippocampal PKMζ inhibition does not appear to significantly affect dormant spatial memory in the MWM (Hales et al., 2016). However, whether this kinase contributes to spatial memory persistence by supporting passive maintenance, reconsolidation, or both, remains an open question.

Here, we confirm that hippocampal PKMζ activity is not required for maintaining inactive spatial memory in the MWM and demonstrate that non-reinforced reactivation renders this memory susceptible to disruption by the PKMζ inhibitor myristoylated ζ-inhibitory peptide (ZIP). This disruption was both long-lasting and time-dependent, and required GluN2B-NMDAR signaling, proteasome activity, and AMPAR endocytosis. A similar amnesic effect was observed following transient hippocampal PKMζ knockdown, but not after PKCι/λ inhibition. Notably, ZIP did not impair spatial memory retention when administered without memory reactivation or following reinforced reactivation. Taken together, our findings indicate that hippocampal PKMζ is essential for reconsolidation of spatial memory in the MWM but dispensable for preserving it while in a quiescent, inactive state.

## Materials and methods

### Animals

We used a total of 285 male Wistar rats, three-month-old and weighing 300–350 g. The animals were housed in groups of 4–5 per cage in ventilated racks, with ad libitum access to food and water. The animal facility maintained a controlled environment with temperatures ranging from 22 to 23°C and a standard 12-h light/dark cycle (lights on at 6:00 a.m., lights off at 6:00 p.m.). Naïve littermates were randomly assigned to experimental groups. All procedures were performed in accordance with the National Institutes of Health (NIH) Guide for the Care and Use of Laboratory Animals and followed the ARRIVE guidelines. The study protocol was approved by the local ethics committee (Comissão de Ética no Uso de Animais, Universidade Federal do Rio Grande do Norte).

### Surgical procedures

Stereotaxic surgeries were performed under anesthesia with ketamine (80 mg/kg) and xylazine (10 mg/kg). Coordinates were determined relative to Bregma, based on previous reports (Luft et al., 2004; Rossato et al., 2007, 2010). Stainless steel guide cannulas (22-G) were bilaterally implanted into the CA1 region of the dorsal hippocampus at the following coordinates: AP −4.2 mm, LL ± 3.0 mm, and DV −2.0 mm (Paxinos and Watson, 2007). All implants were secured to the skull with auto-polymerizing dental resin. After surgery, the animals were administered meloxicam (0.2 mg/kg) subcutaneously as an analgesic and were allowed to recover for 7-d before any other procedure.

### Drug administration

Myristoylated ζ-inhibitory peptide (ZIP), its scrambled inactive control (scZIP), RO25-6981 (RO), and Pep2m (PEP) were from Tocris Bioscience or FastBIO. PKMζ antisense (ASO; 5′-CTCTTGGGAAGGCATGA-3′; 2 nmol/μl) and PKMζ missense oligonucleotides (MSO; 5′-AACAATGGGTCGTCTCG-3′; 2 nmol/μl) phosphorothioated on the three terminal bases to avoid nuclease degradation, were from GBT-Oligos. Dynasore hydrate (DYN) and clasto-lactacystin β-lactone (LAC) were from Sigma-Aldrich. ICAP was a generous gift from Dr. Robert V. Farese (University of South Florida). Drugs were prepared following the manufacturer’s guidelines and stored at −20°C, shielded from light, until needed. Doses were determined based on prior studies. Intra-dCA1 infusions were performed using injectors that extended 1 mm beyond the guide cannulas. The injectors were connected to Hamilton syringes via polyethylene tubes. On the day of the experiment, stock aliquots were thawed and diluted to the desired concentration using sterile saline (VEH). Injections (1 μL per side, delivered at a rate of 0.5 μL/min) were administered with Hamilton syringes connected to infusion pumps (Harvard Apparatus). To confirm accurate infusion placement, 4% methylene blue (1 μL) was injected into dorsal CA1. Animals were then euthanized, and their brains were extracted and analyzed for dye diffusion, which helped verify the spread of the drug or vehicle. Nine animals with misplaced cannula implants were excluded from statistical analysis.

### Morris water maze procedures and data analysis

The MWM apparatus consisted of a 200-cm diameter circular black pool made of brick and concrete. For data analysis the pool was conceptually divided into four quadrants. It was in a well-lit room featuring multiple distal cues on the walls to support spatial navigation (Bonini et al., 2007; da Silva et al., 2012; Gonzalez et al., 2019). A removable plastic partition separated this room from an adjacent area that housed the computer system and temporary accommodations for the rats. A 12-cm diameter escape platform with a rough surface was submerged 2-cm below the water’s surface. The water was maintained at 23°C and rendered opaque using a non-toxic, flavorless, and odorless dye. Ceiling-mounted cameras tracked and recorded the animals’ swimming paths, which were later analyzed using TopScan (CleverSys Inc.). All procedures adhered to NIH guidelines and were approved by the local ethics committee (Comissão de Ética no Uso de Animais, UFRN).

Before training began, rats were handled for 5-min per day over 3 consecutive days. Training consisted of 8 trials per day for 7-d. The escape platform remained fixed in position, while the starting points varied across trials. Each trial ended with a 30-s stay on the escape platform. To reactivate spatial memory and induce reconsolidation, MWM-trained rats underwent a 60-s probe trial without the escape platform at different time points after the final training session. Control animals either did not undergo reactivation or were exposed to a reinforced probe trial. To evaluate treatment effects on the persistence of reactivated memory, a second, non-reinforced probe trial was conducted at various intervals following the first. To verify that the memory being assessed was spatial in nature, a subset of MWM-trained rats underwent a probe trial in which the maze was surrounded by a white matte plastic curtain that blocked all distal cues. Each animal completed a single training-reactivation-testing cycle.

Time in the target quadrant was defined as the percentage of the 60-s probe trial spent swimming within the virtual quadrant where the escape platform was located during training (25% of the maze area). Time in the critical zone referred to the percentage of the 60-s probe trial time spent in a 32-cm diameter virtual circle (2.5% of maze area) centered on the original platform location.

Search entropy was calculated based on information theory principles, where entropy quantifies the uncertainty of a random variable. In this context, search entropy (H) is defined as the sum of two components: error entropy ( $H_{\text{error}})$ , representing the variance of the rat’s position relative to the escape platform, and path entropy ( $H_{\text{path}})$ , representing the variance relative to the focal point of the rat’s swimming trajectory, as described by Maei et al. (2009). This measure captures the progression from disorganized, high-entropy search patterns to more focused, low-entropy behavior as learning occurs. Entropy was computed using the formula: $H=H_{\text{error}}+H_{\text{path}}=\ln(\sigma_{d}^{2})+\ln(\sigma_{a}\sigma_{b})$ . Here, $\sigma_{x}$ denotes the radii of the major axes of the error ellipse. These computations were carried out using a custom Python 3.9 script built with NumPy, SciPy, and Matplotlib libraries. For a full derivation of the entropy algorithm (see Maei et al., 2009). To generate density plots, trajectory files were normalized in scale and proportion to a common circular reference geometry. This normalization included correcting for camera distortion along the x-axis, adjusting the apparent maze radius, and re-centering the coordinate origin to ensure comparability across behavioral sessions and experimental conditions. To reduce edge artifacts in the density estimation, an annular ring of artificial points was added along the maze’s inner circumference. A two-dimensional kernel density estimation was then applied to the combined dataset. Each data point was assigned a Gaussian kernel, with bandwidth determined automatically. The superposition of these kernels produced a continuous occupancy probability surface. Values outside the maze’s physical boundaries were masked, so the final output represented only the animal-accessible space. The result was a smoothed heatmap, free from edge artifacts, indicating areas of high and low dwell time throughout the trial. The corresponding plot includes semi-transparent black traces of the animal’s trajectory, the maze outline, and a blue circle marking the submerged platform’s location and size.

### Statistical analyzes

Statistical analyzes were performed using GraphPad Prism 10 and RStudio. Data were analyzed using either one-way, two-way mixed-design, or three-way mixed-design ANOVA followed by Bonferroni *post-hoc* comparisons, as appropriate. In some cases, Student’s *t*-test or one sample Student’s *t*-test was used. Normality was assessed using D’Agostino-Pearson or Shapiro–Wilk tests. For the experiments requiring a repeated measures analysis, rather than assuming sphericity, we applied the Greenhouse and Geisser correction, which provides a more cautious test of significance, particularly when the assumption of sphericity is violated. The significance level was set at α = 0.05, and sample sizes were determined based on previous studies. In the figures, data points represent individual subjects within each experimental group. Researchers were blinded to the treatment conditions during both data collection and analysis.

## Results

**Figure 1:**
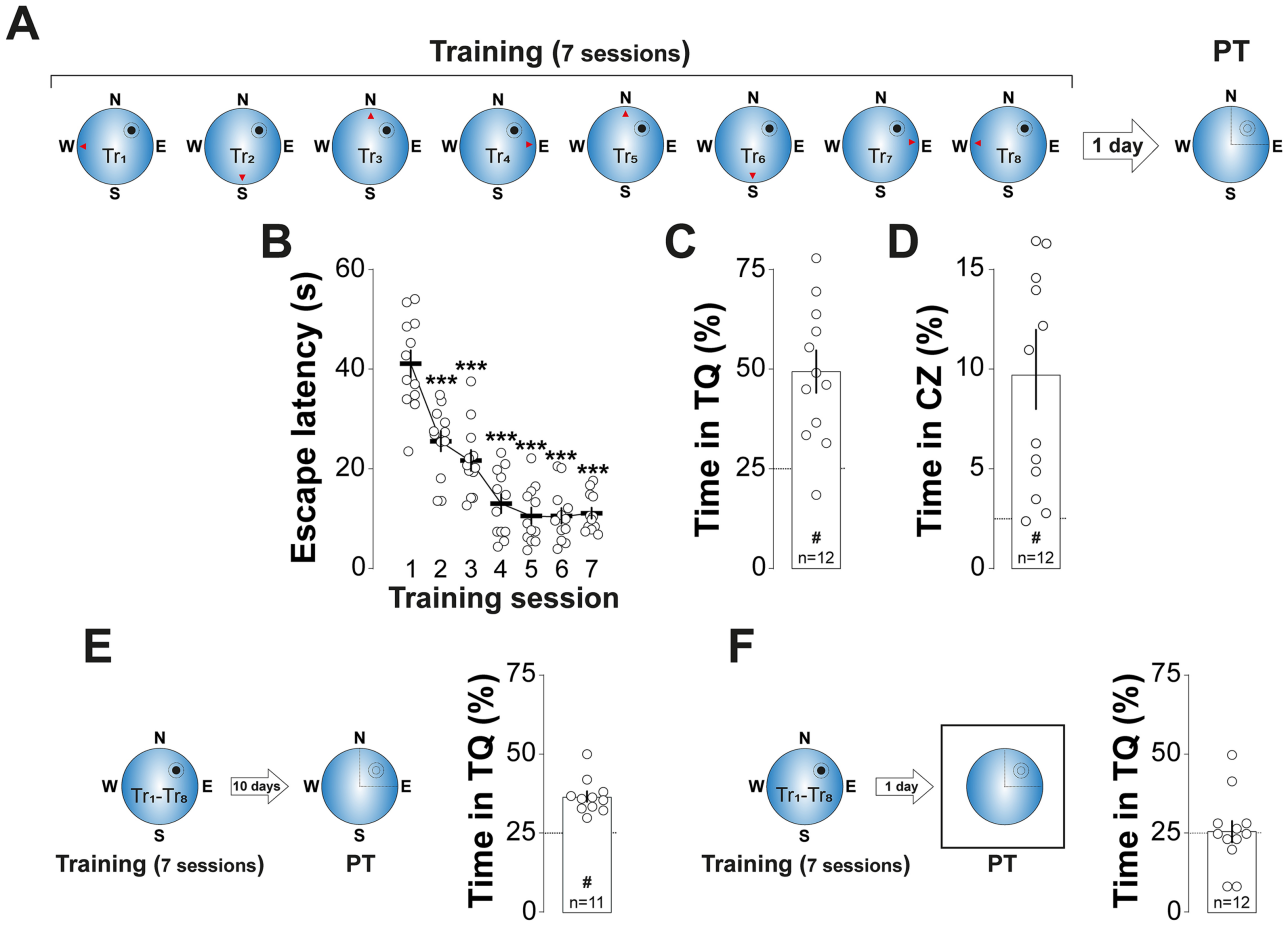
MWM training induces long-lasting spatial preference. **(A)** *Experimental protocol:* Rats underwent daily training sessions in the spatial version of the MWM for 7 consecutive days. Each session consisted of 8 trials (Tr1-Tr8). The red arrowheads indicate the starting positions for each trial, while the black circle and the surrounding circumference mark the location of the escape platform and the critical zone, respectively. Spatial memory retention was assessed 1-d after the final training session with a probe trial (PT), in which the escape platform was removed. **(B)** Escape latency (i.e., the time taken to locate and climb onto the escape platform) is shown as a function of training session. Each dot represents the mean scape latency across the 8 trials of each session for each animal. **(C)** Time spent in the target quadrant (TQ) during PT expressed as a percentage of the total session time for the animals shown in **(B)**. ^#^*p* < 0.01 in one sample *t*-test against a theoretical mean of 25. **(D)** Time spent in the critical zone (CZ) during PT expressed as a percentage of the total session time for the animals shown in **(B)**. ^#^*p* < 0.01 in one sample *t*-test against a theoretical mean of 2.5. **(E)** *Left panel:* Experimental protocol. Rats were trained as described in **(A)** but PT was conducted 10-d after the last training session. *Right panel:* Time spent in TQ during PT, expressed as a percentage of the total session time. ^#^*p* < 0.05 in one sample *t*-test against a theoretical mean of 25. **(F)** *Left panel:* Experimental protocol. Rats were trained as described in **(A)** but during PT, conducted 1-d after the last training session, the maze was surrounded by a white matte plastic curtain blocking all distal cues. *Right panel:* Time spent in TQ during PT, expressed as a percentage of the total session time. Data are presented as mean ± SEM with *n* = 11–12 animals per group. Dashed lines represent chance levels. **p < 0.01 in Bonferroni’s multiple comparison test following repeated measures one-way ANOVA.

The MWM training protocol employed in our study (Figure 1A) resulted in robust spatial learning, as demonstrated by a progressive decrease in escape latency across training sessions (Figure 1B; *F*(3.455, 38.01) = 50.06, *p* < 0.001, in repeated measures one-way ANOVA with Greenhouse and Geisser correction) and a strong preference for swimming in the target quadrant (Figure 1C; *t*(11) = 4.606, *p* < 0.001, in one sample *t*-test), especially within a 32-cm diameter critical zone centered on the previous location of the escape platform (Figure 1D; *t*(11) = 3.637, *p* < 0.01, in one sample *t*-test), during a 60-s, non-reinforced probe trial conducted 1-d after training. This spatial preference persisted for at least 10-d post-training (Figure 1E; *t*(10) = 7.118, *p* < 0.001, in one sample *t*-test), but was abolished when distal spatial cues were removed during the probe trial (Figure 1F), confirming the spatial specificity of the memory under investigation.

**Figure 2:**
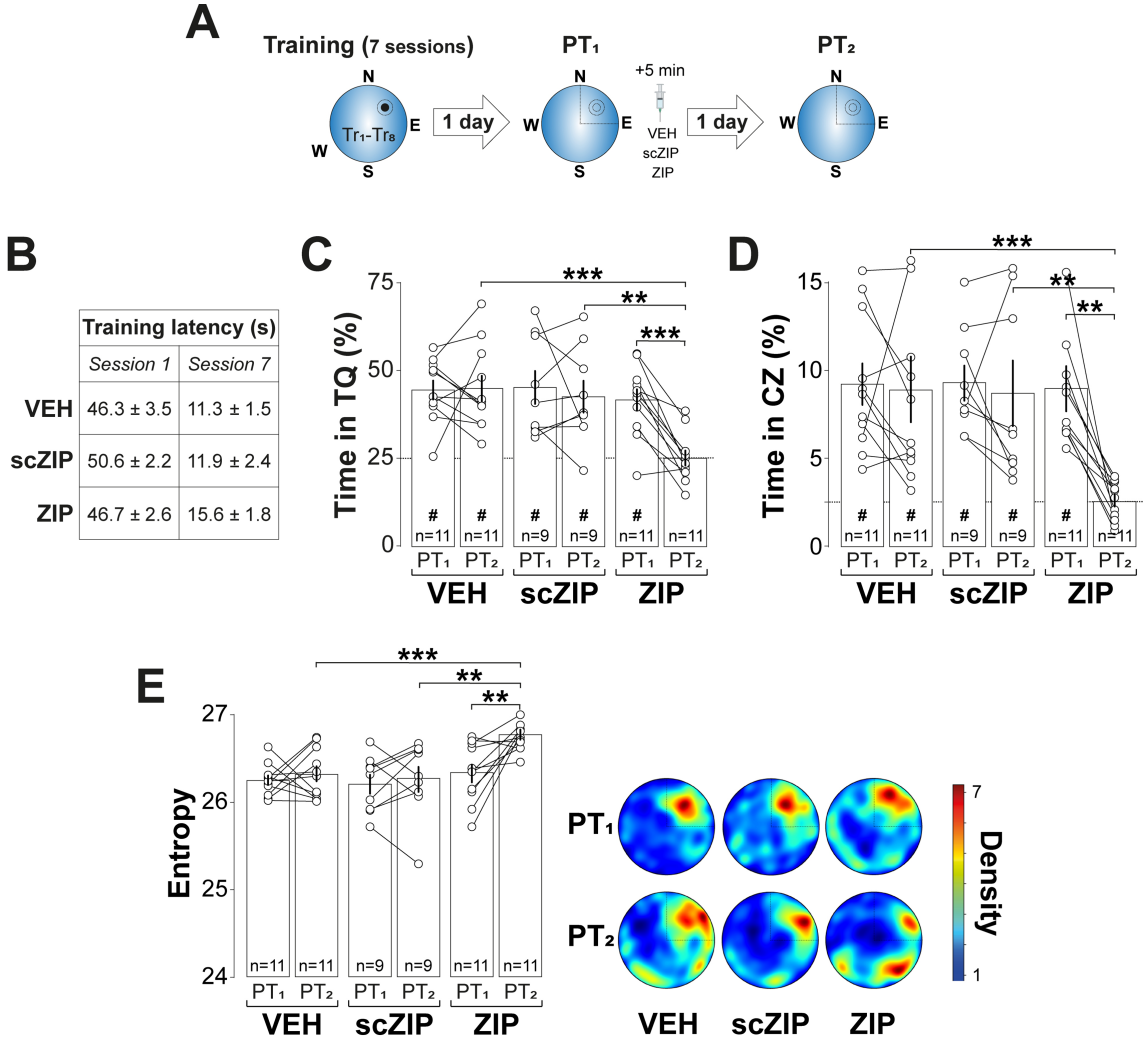
Intra-dorsal CA1 ZIP impairs reactivated spatial memory retention. **(A)** *Experimental protocol:* Rats were trained daily in the spatial version of the MWM for 7 consecutive days, with each session consisting of 8 trials (Tr1-Tr8). Memory was reactivated 1-d after the final training session using a non-reinforced probe trial (PT1). Rats then received bilateral intra-dCA1 infusions of vehicle (VEH), scZIP, or ZIP, administered 5-min post-PT1. Retention was assessed 1-d after PT1 with a second non-reinforced PT (PT2). **(B)** Mean escape latency for the 3 experimental groups during the first (Session 1) and the final (Session 7) training sessions. **(C)** Time spent in the target quadrant (TQ) during PT1 and PT2 expressed as a percentage of the total session time; ^#^*p* < 0.01 in one sample *t*-test against a theoretical mean of 25. **(D)** Time spent in the critical zone (CZ) during PT1 and PT2 expressed as a percentage of the total session time; ^#^*p* < 0.05 in one sample *t*-test against a theoretical mean of 2.5. **(E)** *Left panel*: Search entropy during PT1 and PT2. *Right panel*: Average heat maps of position density for each experimental group during PT1 and PT2. Data are presented as mean ± SEM with *n* = 9–11 animals per group. Dashed lines represent chance levels. **p < 0.01 and ***p < 0.001 in Bonferroni’s multiple comparison test following two-way mixed-design ANOVA.

To examine the role of hippocampal PKMζ in spatial memory reconsolidation, we infused the PKMζ inhibitor myristoylated ζ-inhibitory peptide (ZIP; 1 nmol/μl; Ling et al., 2002) or its scrambled inactive control (ScZIP; 1 nmol/μl) into the CA1 region of the dorsal hippocampus (dCA1) 5-min after a 60-s non-reinforced probe trial conducted 1-d after the final MWM training session (PT1). Due to concerns about ZIP’s specificity, since standard concentrations have been shown to impair plasticity even in PKMζ knockout mice (Volk et al., 2013), disrupt brain oscillations (LeBlancq et al., 2016), and interfere with CaMKII and PKCι/*λ* activity (Kwapis and Helmstetter, 2014), we administered a dose ten times lower than typically used. This reduced concentration effectively inhibits PKMζ without affecting hippocampal oscillations, multi-unit activity, or CaMKII autophosphorylation (Rossato et al., 2019). Rats that received ZIP, but not those given scZIP, exhibited a significant reduction in time spent in the target quadrant (Figure 2C; *F*(2, 28) = 5.028, *p* = 0.0136 for Treatment; *F*(1, 28) = 8.324, *p* = 0.0074 for PT session; *F*(2, 28) = 6.116, *p* = 0.0063 for Treatment x PT session, in two-way mixed-design ANOVA) and in the critical zone (Figure 2D; *F*(2, 28) = 3.401, *p* = 0.0476 for Treatment; *F*(1, 28) = 6.542, *p* = 0.0162 for PT session; *F*(2, 28) = 5.099, *p* = 0.0129 for Treatment x PT session, in two-way mixed-design ANOVA), along with increased search entropy (Figure 2E; *F*(2, 28) = 7.062, *p* = 0.0033 for Treatment; *F*(1, 28) = 6.283, *p* = 0.0183 for PT session; *F*(2, 28) = 3.402, p = 0.0476 for Treatment x PT session, in two-way mixed-design ANOVA) during a second probe trial conducted 1-d later (PT2).

**Figure 3:**
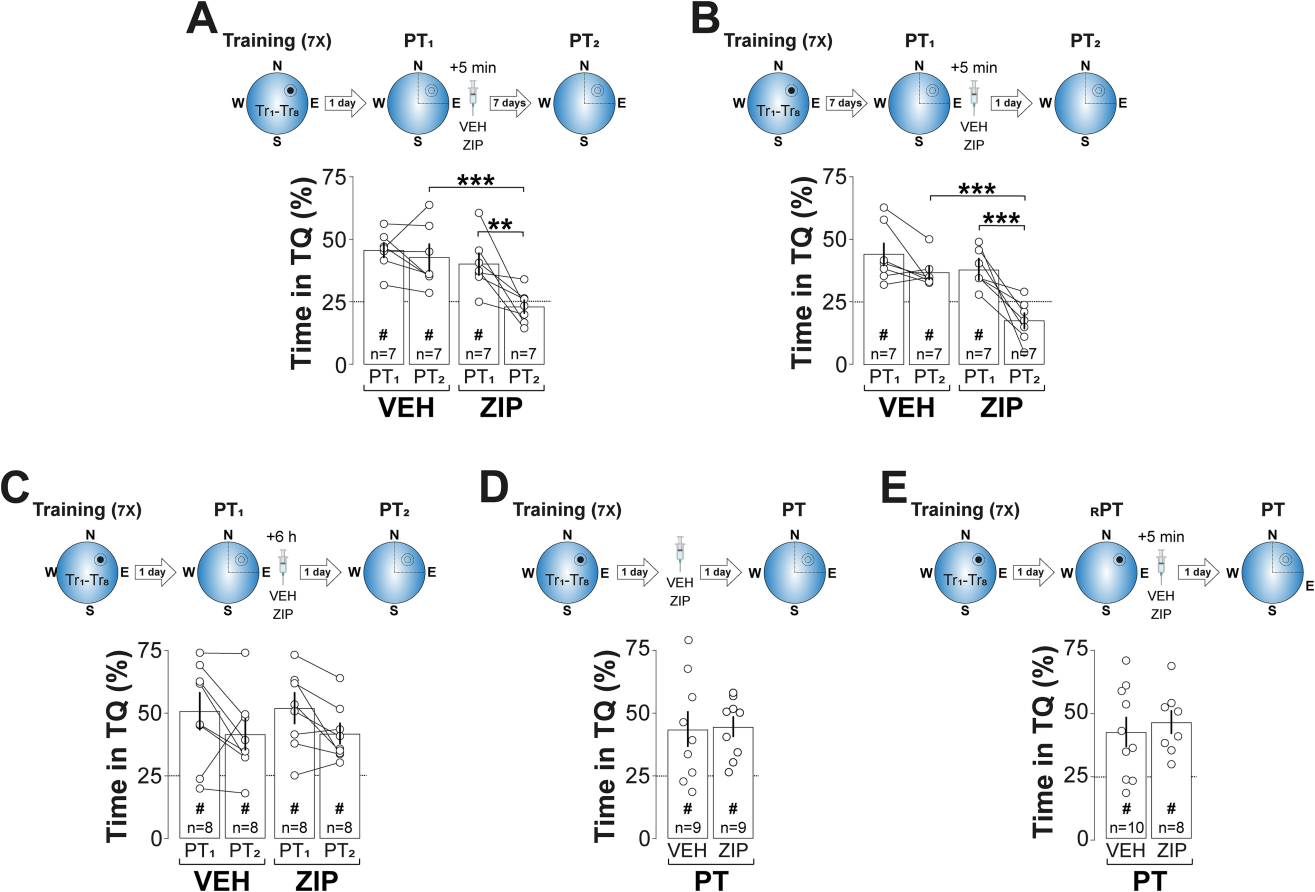
ZIP-induced amnesia is long-lasting, time-dependent, memory age-independent, and selective to non-reinforced reactivation. **(A)** *Upper panel:* Experimental protocol. Rats were trained daily in the spatial version of the MWM for 7 consecutive days, with each session consisting of 8 trials (Tr1-Tr8). Memory was reactivated 1-d after the final training session using a non-reinforced probe trial (PT1). Rats then received bilateral intra-dCA1 infusions of vehicle (VEH) or ZIP, administered 5-min post-PT1. Retention was assessed 7-d later with a second non-reinforced PT (PT2). *Lower panel:* Time spent in the target quadrant (TQ) during PT1 and PT2, expressed as a percentage of the total session time. **(B)** *Upper panel:* Experimental protocol. Rats were trained and treated as described in **(A)**, but memory was reactivated 7-d after the final training session, with retention assessed 1-d afterward. *Lower panel:* Time spent in TQ during PT1 and PT2, expressed as a percentage of the total session time. **(C)** *Upper panel:* Experimental protocol. Rats were trained and memory was reactivated as described in **(A)**, but the animals received bilateral intra-dCA1 infusions of VEH or ZIP 6-h post-PT1. Retention was assessed 1-d later. *Lower panel:* Time spent in TQ during PT1 and PT2, expressed as a percentage of the total session time. **(D)** *Upper panel:* Experimental protocol. Rats were trained as described in **(A)** and received bilateral intra-dCA1 infusions of VEH or ZIP 1-d afterwards. Retention was assessed 1-d later with a non-reinforced PT. *Lower panel:* Time spent in TQ during PT, expressed as a percentage of the total session time. **(E)** *Upper panel:* Experimental protocol. Rats were trained as described in **(A)**. Memory was reactivated 1-d after the final training session using a reinforced PT (_R_PT). Rats then received bilateral intra-dCA1 infusions of VEH or ZIP, administered 5-min post-_R_PT. Retention was assessed 1-d later with a non-reinforced PT. *Lower panel:* Time spent in TQ during PT, expressed as a percentage of the total session time. ^#^*p* < 0.05 in one sample *t*-test against a theoretical mean of 25. Data are presented as mean ± SEM with *n* = 7–10 animals per group. Dashed lines represent chance levels. **p < 0.01 and ***p < 0.001 in Bonferroni’s multiple comparison test following two-way mixed-design ANOVA.

**Figure 4:**
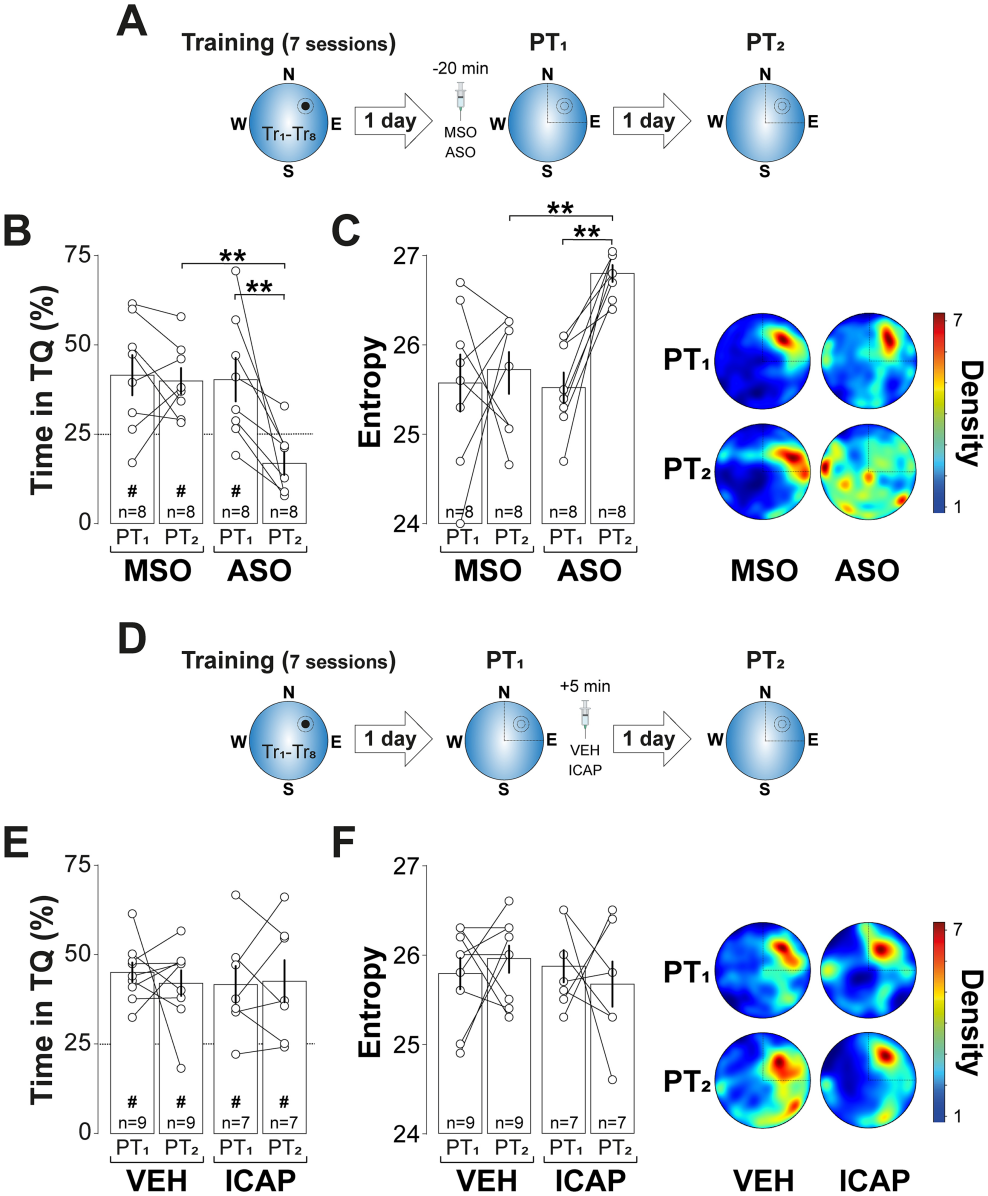
PKMζ silencing, but not PKCι/*λ* inhibition, disrupts reactivated spatial memory retention. **(A)** *Experimental protocol:* Rats were trained daily in the spatial version of the MWM for 7 consecutive days, with each session consisting of 8 trials (Tr1-Tr8). Memory was reactivated 1-d after the final training session using a non-reinforced probe trial (PT1). Twenty min before PT1 rats received bilateral intra-dCA1 infusions of phosphorothioated antisense oligonucleotides against PKMζ (ASO) or missense control oligonucleotides (MSO). Retention was assessed 1-d later with a second non-reinforced PT (PT2). **(B)** Time spent in the target quadrant (TQ) during PT1 and PT2, expressed as a percentage of the total session time. **(C)** *Left panel:* Search entropy during PT1 and PT2. *Right panel:* Average heat maps of position density for each experimental group during PT1 and PT2. **(D)** *Experimental protocol:* Rats were trained, and memory was reactivated as described in **(A)**, but the animals received bilateral intra-dCA1 infusions of vehicle (VEH) or ICAP 5-min post-PT1. **(E)** Time spent in TQ during PT1 and PT2, expressed as a percentage of the total session time. **(F)** *Left panel:* Search entropy during PT1 and PT2. *Right panel:* Average heat maps of position density for each experimental group during PT1 and PT2. ^#^*p* < 0.05 in one sample *t*-test against a theoretical mean of 25. Data are presented as mean ± SEM with *n* = 7–9 animals per group. Dashed lines represent chance levels. **p < 0.01 in Bonferroni’s multiple comparison test following two-way mixed-design ANOVA.

The amnesic effect of ZIP persisted for at least 7-d (Figure 3A; *F*(1, 12) = 9.122, *p* = 0.0107 for Treatment; *F*(1, 12) = 9.935, *p* = 0.0083 for PT session; *F*(1, 12) = 5.063, *p* = 0.044 for Treatment x PT session, in two-way mixed-design ANOVA) and was also observed when spatial memory was reactivated 7-d, rather than 1-d, after training (Figure 3B; *F*(1, 12) = 14.47, *p* = 0.0025 for Treatment; *F*(1, 12) = 24.41, *p* = 0.0003 for PT session; *F*(1, 12) = 5.427, *p* = 0.0381 for Treatment vs. PT session, in two-way mixed-design ANOVA). In contrast, ZIP had no effect on retention when administered 6-h post-reactivation (Figure 3C), in the absence of reactivation (Figure 3D), or following a reinforced probe trial in which the escape platform remained in its original training location (Figure 3E). Silencing PKMζ in dCA1 using phosphorothioated antisense oligonucleotides (Figure 4A; ASO; 5′-CTCTTGGGAAGGCATGA-3′; 2 nmol/μl), but not missense control oligonucleotides (MSO; 5′-AACAATGGGTCGTCTCG-3′; 2 nmol/μl), produced amnesia comparable to that induced by ZIP. This was demonstrated by significant reduction in time spent in the target quadrant (Figure 4B; *F*(1, 14) = 4.957, *p* = 0.0429 for Treatment; *F*(1, 14) = 9.525, p = 0.008 for PT session; *F*(1, 14) = 7.243, *p* = 0.0176 for Treatment x PT session, in two-way mixed-design ANOVA) and increased search entropy (Figure 4C; *F*(1, 14) = 8.269, *p* = 0.0122 for Treatment; *F*(1, 14) = 7.781, *p* = 0.0145 for PT session; *F*(1, 14) = 4.849, *p* = 0.0449 for Treatment x PT session, in two-way mixed-design ANOVA). Conversely, post-reactivation inhibition of dCA1 PKCι/*λ* with ICAP (1 nmol/μl; Tsokas et al., 2016) did not impact retention (Figures 4D–F).

**Figure 5:**
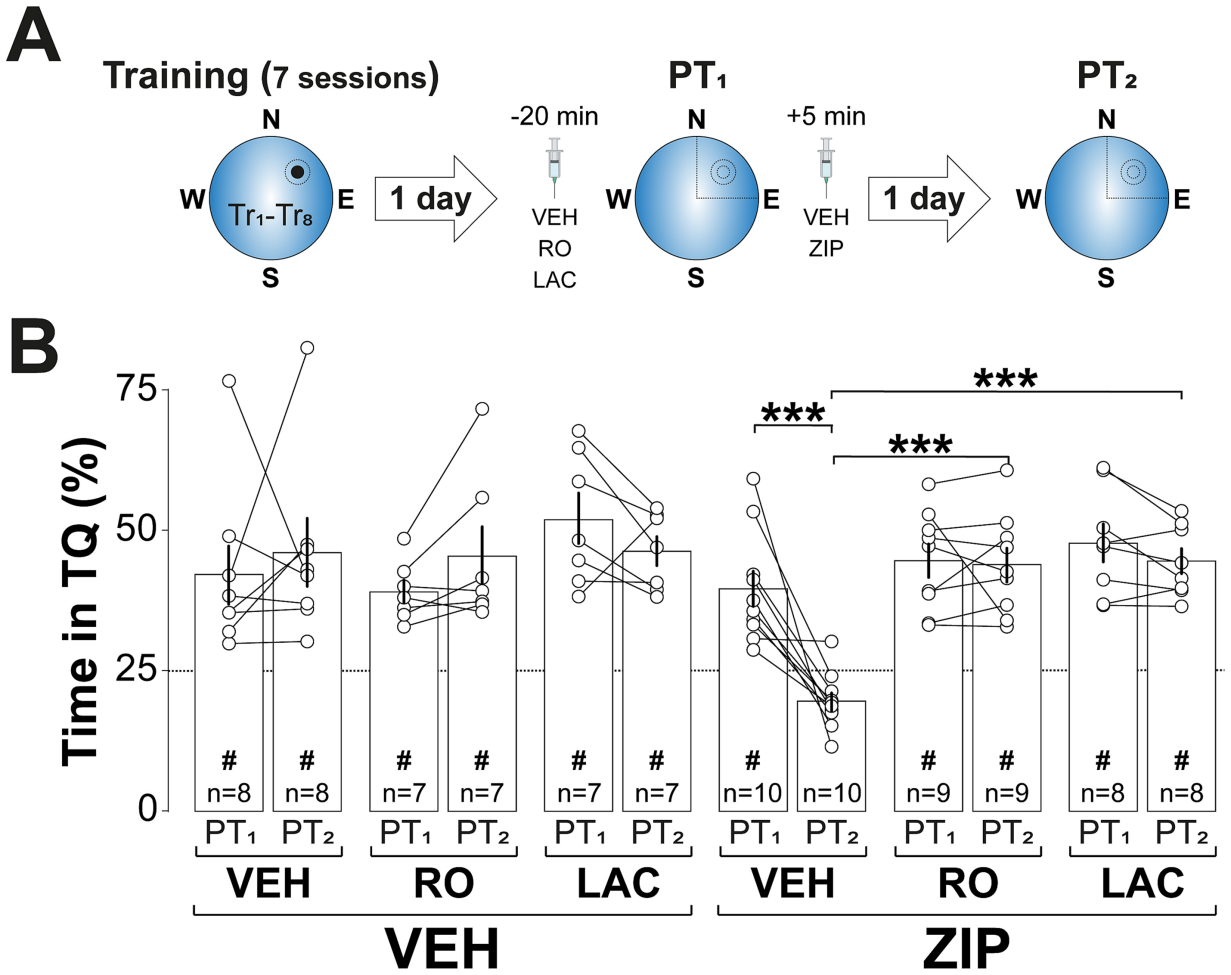
Inhibiting memory destabilization by blocking GluN2B-NMDAR signaling or proteasome activity prevents ZIP-induced amnesia. **(A)** *Experimental protocol:* Rats were trained daily in the spatial version of the MWM for 7 consecutive days, with each session consisting of 8 trials (Tr1-Tr8). Memory was reactivated 1-d after the final training session using a non-reinforced probe trial (PT1). Twenty min before PT1 rats received bilateral intra-dCA1 infusions of vehicle (VEH), RO25-6981 (RO), or clasto-lactacystin β-lactone (LAC) and 5-min after that session were given either VEH or ZIP. Retention was assessed 1-d later with a second non-reinforced PT (PT2). **(B)** Time spent in the target quadrant (TQ) during PT1 and PT2, expressed as a percentage of the total session time. ^#^*p* < 0.05 in one sample *t*-test against a theoretical mean of 25. Data are presented as mean ± SEM with *n* = 7–10 animals per group. Dashed line represents chance levels. ***p < 0.001 in Bonferroni’s multiple comparison test following three-way mixed-design ANOVA.

Building upon previous findings that GluN2B-NMDARs and proteasome activity are crucial for reactivated memory destabilization (Milton et al., 2013; Lee and Flavell, 2014; Radiske et al., 2021, 2023; Rossato et al., 2023), and necessary for rendering contextual fear memories susceptible to amygdalar PKMζ silencing after recall (Bernabo et al., 2021), we hypothesized that blocking these processes in dCA1 would prevent ZIP-induced spatial memory amnesia. Consistent with this hypothesis, intra-dCA1 infusion of either the GluN2B-NMDAR antagonist RO25-6981 (RO; 2.5 μg/side; Fischer et al., 1997) or the proteasome inhibitor clasto-lactacystin β-lactone (LAC; 3.2 ng/side; Lee et al., 2008) 20-min before reactivation ((Figure 5A) did not impair spatial memory recall but effectively blocked the amnesia induced by post-reactivation ZIP administration (Figure 5B; *F*(2, 43) = 6.964, 0.0024 for Pre-PT1 treatment; *F*(1, 43) = 4.581, *p* = 0.038 for Post-PT1 treatment; *F*(2, 43) = 3.37, *p* = 0.0437 for Pre-PT1 treatment x PT session; *F*(2, 43) = 4.37, *p* = 0.0187 for Pre-PT1 treatment x Post-PT1 treatment; *F*(1, 43) = 7.267, p = 0.01 and *F*(2, 43) = 4.852, *p* = 0.0126 for Pre-PT1 treatment x PT session x Post-PT1 treatment, in three-way mixed-design ANOVA).

**Figure 6:**
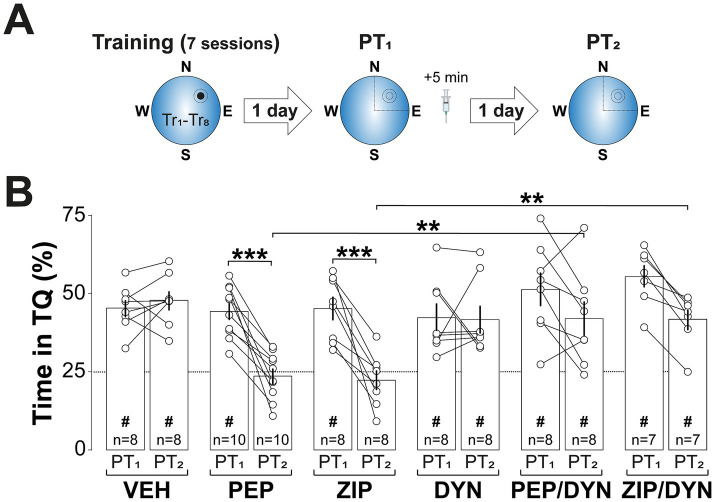
Inhibiting AMPAR endocytosis reverses ZIP-induced amnesia. **(A)** *Experimental protocol:* Rats were trained daily in the spatial version of the MWM for 7 consecutive days, with each session consisting of 8 trials (Tr1-Tr8). Memory was reactivated 1-d after the final training session using a non-reinforced probe trial (PT1). Five min after PT1 rats received bilateral intra-dCA1 infusions of vehicle (VEH), Pep2m (PEP), ZIP, DYN, or a combination of either PEP and DYN or ZIP and DYN. Retention was assessed 1-d later with a second non-reinforced PT (PT2). **(B)** Time spent in the target quadrant (TQ) during PT1 and PT2, expressed as a percentage of the total session time. ^#^*p* < 0.05 in one sample *t*-test against a theoretical mean of 25. Data are presented as mean ± SEM with *n* = 7–10 animals per group. Dashed line represents chance levels. **p < 0.01 and ***p < 0.001 in Bonferroni’s multiple comparison test following two-way mixed-design ANOVA.

PKMζ is known to maintain synaptic AMPAR levels by inhibiting N-ethylmaleimide-sensitive factor (NSF)-mediated endocytosis (Yao et al., 2008), a mechanism essential for both LTP and memory maintenance (Migues et al., 2014; Dong et al., 2015) and implicated in memory reconsolidation (Rossato et al., 2019; Bernabo et al., 2021). In line with this, Pep2m (PEP; 5 pmol/μl; Lüthi et al., 1999), a peptide that disrupts NSF/GluA2 interaction and reduces surface AMPARs expression (Ralph et al., 2001), induced spatial memory amnesia when administered into dCA1 5-min after non-reinforced spatial memory reactivation. In contrast, dynasore hydrate (DYN; 120 pmol/μl; Kirchhausen et al., 2008; Figure 6A), a cell-permeable dynamin inhibitor that blocks AMPAR internalization (Ferreira et al., 2015) and increases synaptic expression of GluA1/2-containing AMPAR (Rossato et al., 2019), reversed the amnesia caused by both Pep2m and ZIP (Figure 6B; *F*(5, 43) = 4.879, *p* = 0.0013 for Treatment; *F*(1, 43) = 44.77, *p* < 0.001 for Test session; *F*(5, 43) = 7.098, *p* < 0.001 for Treatment x PT session, in two-way mixed-design ANOVA). Table 1 presents the one-sample *t*-test results comparing animal performance against chance levels for Figure 2 through Figure 6.

**Table 1:** One-sample *t*-test results comparing animal performance against chance levels for Figure 2 through Figure 6.

	Treatment	Behavioral session	*t* value	*p* value
Figure 2C	VEH	PT1	*t*(10) = 7.333	<0.0001
		PT2	*t*(10) = 5.527	=0.0003
	scZIP	PT1	*t*(8) = 4.354	=0.0024
		PT2	*t*(8) = 3.902	=0.0045
	ZIP	PT1	*t*(10) = 5.528	<0.0001
		PT2	*t*(10) = 0.099	=0.9232
Figure 2D	VEH	PT1	*t*(10) = 7.848	<0.0001
		PT2	*t*(10) = 4.790	=0.0007
	scZIP	PT1	*t*(8) = 9.352	<0.0001
		PT2	*t*(8) = 4.679	=0.0016
	ZIP	PT1	*t*(10) = 6.974	<0.0001
		PT2	*t*(10) = 7.650	<0.0001
Figure 3A	VEH	PT1	*t*(6) = 7.109	=0.0004
		PT2	*t*(6) = 3.692	=0.0102
	ZIP	PT1	*t*(6) = 3.639	=0.0108
		PT1	*t*(6) = 0.801	=0.4537
Figure 3B	VEH	PT1	*t*(6) = 4.347	=0.0048
		PT2	*t*(6) = 5.101	=0.0022
	ZIP	PT1	*t*(6) = 6.022	=0.0009
		PT1	*t*(6) = 2.467	=0.0486
Figure 3C	VEH	PT1	*t*(7) = 3.660	=0.0081
		PT2	*t*(7) = 2.885	=0.0235
	ZIP	PT1	*t*(7) = 4.616	=0.0024
		PT1	*t*(7) = 4.492	=0.0028
Figure 3D	VEH	PT	*t*(8) = 2.739	=0.0255
	ZIP	PT	*t*(8) = 5.125	=0.0009
Figure 3E	VEH	PT	*t*(9) = 3.131	=0.0121
	ZIP	PT	*t*(7) = 4.897	=0.0018
Figure 4B	MSO	PT1	*t*(7) = 2.929	=0.0220
		PT2	*t*(7) = 4.127	=0.0044
	ASO	PT1	*t*(7) = 2.499	=0.0410
		PT2	*t*(7) = 2.536	=0.0389
Figure 4E	VEH	PT1	*t*(8) = 7.274	<0.0001
		PT2	*t*(8) = 4.638	=0.0017
	ICAP	PT1	*t*(6) = 3.109	=0.0209
		PT2	*t*(6) = 2.874	=0.0283
Figure 5B	VEH + VEH	PT1	*t*(7) = 3.318	=0.0128
		PT2	*t*(7) = 3.488	=0.0102
	VEH + RO	PT1	*t*(6) = 7.044	=0.0004
		PT2	*t*(6) = 4.022	=0.0069
	VEH + LAC	PT1	*t*(6) = 6.044	=0.0009
		PT2	*t*(6) = 8.288	=0.0002
	ZIP + VEH	PT1	*t*(9) = 4.714	=0.0011
		PT2	*t*(9) = 3.366	=0.0083
	ZIP + RO	PT1	*t*(8) = 6.675	=0.0002
		PT2	*t*(8) = 6.310	=0.0002
	ZIP + LAC	PT1	*t*(7) = 6.742	=0.0003
		PT2	*t*(7) = 8.736	<0.0001
Figure 6B	VEH	PT1	*t*(7) = 8.041	<0.0001
		PT2	*t*(7) = 7.956	<0.0001
	PEP	PT1	*t*(9) = 7.415	<0.0001
		PT2	*t*(9) = 0.563	=0.5871
	ZIP	PT1	*t*(7) = 5.676	=0.0008
		PT2	*t*(7) = 0.894	=0.4008
	DYN	PT1	*t*(7) = 4.129	=0.0044
		PT2	*t*(7) = 3.937	=0.0056
	PEP + DYN	PT1	*t*(7) = 5.075	=0.0014
		PT2	*t*(7) = 3.190	=0.0153
	ZIP + DYN	PT1	*t*(6) = 8.961	=0.0001
		PT2	*t*(6) = 5.482	=0.0015

## Discussion

Altogether, our results demonstrate that spatial memory reconsolidation in the MWM requires PKMζ activity in dCA1, whereas the maintenance of inactive spatial memory does not. This conclusion is supported by the observation that ZIP impaired memory retention in a long-lasting, time-dependent manner only when injected into dCA1 following non-reinforced reactivation, a condition known to destabilize memory in this task (Rossato et al., 2006, 2015; da Silva et al., 2008). In contrast, ZIP had no effect on memory when administered without reactivation or after reinforced reactivation. The fact that the amnesia induced by ZIP was replicated by PKMζ knockdown but not by PKCι/λ inhibition further strengthens this interpretation.

Reactivation-induced memory destabilization, mediated by GluN2B-NMDAR and proteasome activity, converges on PKMζ-regulated AMPAR endocytosis, establishing a critical molecular axis at the onset of memory reconsolidation (Hong et al., 2013; Ferreira et al., 2015, 2021; Patrick et al., 2023). Our finding that blocking any of these mechanisms prevents ZIP-induced amnesia reinforces the notion that PKMζ is essential for memory reconsolidation but dispensable for the maintenance of dormant spatial memories. This challenges earlier studies suggesting that hippocampal PKMζ inhibition impairs the maintenance of inactive long-term spatial memories in the MWM (Serrano et al., 2008). However, in those studies, ZIP was administered prior to memory reactivation, raising the possibility that the observed amnesia reflected disrupted reconsolidation rather than interference with the storage mechanisms underlying the persistence of quiescent memories (Kwapis and Helmstetter, 2014). Additionally, the behavioral effects reported by Serrano et al. (2008) were modest and based on less reliable parameters. Notably, ZIP did not affect the time animals spent in the target quadrant during the retention test, a standard and widely accepted measure of spatial memory retention (Rogers et al., 2017). Instead, the only statistically significant effect reported was a reduction in the number of crossings over the former platform location, an imprecise and inconsistent indicator of spatial preference when used alone (Vorhees and Williams, 2006). Supporting this, Hales and coworkers showed that intrahippocampal ZIP administration 3 to 6-d post-training had no effect on time spent in the target quadrant or the critical zone up to 7-d later (Hales et al., 2016).

While our data unequivocally demonstrate that hippocampal PKMζ activity is required for reconsolidation but not for the passive maintenance of spatial memory in the MWM, this should not be taken to imply that all forms of spatial memory are similarly affected. For instance, Pastalkova and coworkers reported that hippocampal PKMζ is required to sustain spatial memory in a non-standard multi-trial place avoidance task, possibly via mechanisms supporting LTP maintenance (Pastalkova et al., 2006). However, they administered ZIP 2-h before a memory recall session, using a concentration known to affect not only PKMζ but also CaMKII and baseline oscillatory activity. As with Serrano et al. (2008), this raises concerns about pharmacological specificity and opens the possibility that the observed amnesia resulted from interactions between PKMζ inhibition and memory reactivation, an interpretation not addressed by the authors.

PKMζ inhibition in the hippocampus has also been reported to impair maintenance in the object location memory (OLM) task (Hardt et al., 2010). However, while both MWM and OLM are broadly categorized as spatial memory tasks, they differ significantly in their cognitive demands. The MWM involves spatial navigation and the formation of an allocentric cognitive map, whereas OLM relies on establishing positional object-context-subject contingencies, without requiring navigation through a spatial environment. Successful OLM performance hinges not only on recognizing spatial novelty but also on identifying the objects themselves, thus engaging ORM, which is encoded alongside the OLM trace. Similarly, while MWM performance requires learning the spatial location of a hidden platform, it also demands mastery of the behavioral procedures needed to reach it. However, unlike OLM and ORM, which are both hippocampus-dependent (Broadbent et al., 2009; Clarke et al., 2010; Fan et al., 2010; ILL-Raga et al., 2013; Rossato et al., 2007, 2019, 2025; Trimper et al., 2014; Stackman et al., 2016; Cercato et al., 2016; Lymer et al., 2017; Tanimizu et al., 2018; Cinalli et al., 2020; Gonzalez et al., 2022; Takeda et al., 2025), despite reports to the contrary (Bussey et al., 2000; Forwood et al., 2005; Ainge et al., 2006; Good et al., 2007; Barker and Warburton, 2011), the procedural components of the MWM appear to be independent of the hippocampus function (Squire, 1993; Bannerman et al., 1995), though alternative viewpoints exist (Gerlai, 2001; Inostroza et al., 2011). Future experiments should investigate whether the reactivation-dependent amnesia induced by hippocampal PKMζ inhibition spares these procedural elements.

In any case, the sensitivity of a memory trace to PKMζ inhibition likely depends on several factors, including memory age, strength, and the contextual, emotional, and cognitive conditions during encoding and recall. Therefore, it remains plausible that even seemingly inactive spatial memories could be impaired by PKMζ inhibition, especially if they have been subtly or partially reactivated in ways that do not produce an obvious behavioral output. Indeed, in the MWM, spatial memories are destabilized specifically when reactivation creates a mismatch between expectation and experience, suggesting that their reconsolidation can be triggered by cues that may not always be easily detected. Such covert reactivation could go unnoticed yet still render memories labile and vulnerable to disruption (Gisquet-Verrier and Riccio, 2012; Soeter and Kindt, 2015). The possibility that extended training might have altered the underlying mechanisms of inactive memory maintenance, thereby reducing reliance on hippocampal PKMζ, seems unlikely. For example, Serrano et al. (2008) used an eight-arm radial maze paradigm requiring 6-d of training and found that intra-hippocampal ZIP administration abolished long-term memory maintenance. Likewise, Hardt et al. (2010) and Augereau et al. (2022) used prolonged training protocols with 5 to 10-min daily training sessions over 5 to 7-d to demonstrate that hippocampal PKMζ is necessary for the persistent storage of OLM.

Before concluding, a cautionary note is warranted. As with most studies supporting a role for PKMζ in memory, the experiments presented here relied heavily, though not exclusively, on ZIP. Like any pharmacological agent, ZIP may have off target effects. Although we used a dose 10-times lower than typically employed, it remains possible that ZIP’s effects resulted from mechanisms other than PKMζ inhibition. Importantly, the limitations associated with using ZIP to study PKMζ’s role in spatial memory reconsolidation are not unique to this process; they also apply to other memory phenomena, including consolidation, extinction, and LTP. Nonetheless, it is unlikely that the differential effects of ZIP on inactive versus reactivated spatial memories observed in our study were due to the reduced concentration we used, as reconsolidation is typically more resistant to disruption than consolidation (Debiec et al., 2002). Furthermore, the possibility that the observed amnesia resulted from a general impairment of hippocampal function was ruled out through appropriate control experiments. However, we cannot entirely exclude the alternative explanation that the amnesia induced by post-reactivation ZIP infusion reflects a transient, albeit long-lasting, performance deficit. Unfortunately, methodological constraints related to task design and the welfare of cannulated animals prevented us from testing recall at longer retention intervals. This remains a significant limitation, given that prior research indicates the amnesic effects of reconsolidation blockade may vary with task parameters and, in some cases, be reversible (Riccio et al., 2002; Cammarota et al., 2004; Trent et al., 2015; Prado-Alcalá et al., 2017; Radiske et al., 2017, 2025).

In conclusion, our findings support and extend earlier critiques of PKMζ as a universal mechanism for memory maintenance (Kwapis et al., 2009; Parsons and Davis, 2011; Frankland and Josselyn, 2013; Kwapis and Helmstetter, 2014; Morris, 2016). Rather than indicating a role in the sustained storage of inactive memories, our results point to a more specific involvement in the persistence of memories that have been destabilized through reactivation. This distinction has significant implications for future research, particularly regarding recent memories, which are typically more susceptible to reconsolidation than remote ones (Frankland et al., 2006).

## Data Availability

The raw data supporting the conclusions of this article will be made available by the authors, without undue reservation.
